# Opening the ‘black box’: liaison psychiatry services and what they actually do

**DOI:** 10.1192/pb.bp.115.051771

**Published:** 2016-08

**Authors:** Elspeth Guthrie, Aaron McMeekin, Rachel Thomasson, Sylvia Khan, Sally Makin, Ben Shaw, Damien Longson

**Affiliations:** 1 University of Manchester; 2 Manchester Mental Health and Social Care NHS Trust; 3 Lancashire Care NHS Foundation Trust; 4 Pennine Acute Hospitals NHS Trust; 5 Greater Manchester West Mental Health NHS Foundation Trust

## Abstract

**Aims and method** To develop a simple, pragmatic typology to characterise the nature of liaison interventions delivered by a liaison service in a National Health Service setting. We carried out a retrospective electronic case-note review of referrals to a ward-based liaison psychiatry service.

**Results** Three hundred and forty-four patients were referred to the service over a 12-month period. Ten different types of liaison interventions were identified, with the most common interventions being diagnosis (112 patients, 32.6%), medication management (57 patients, 16.6%), risk assessment and treatment (56 patients, 16.3% each). Mental Health Act work accounted for the greatest number of contacts per patient (median 7).

**Clinical implications** There are inherent limitations in any single-site observational study, as site-specific results cannot be generalised to other liaison services. The intervention categories we developed, however, are easy to use and will provide a way of comparing and benchmarking the range of interventions delivered by different liaison psychiatry services.

In a recent editorial, Sharpe^1^ has argued that liaison psychiatry services need to develop greater clarity about what they are seeking to achieve, with definable and measurable outcomes. Currently, liaison services have rather a ‘black box’ quality in that they are present in hospitals, they potentially save money, they see a lot of patients, but there has been a struggle to capture the range and type of clinical interventions that they provide.

The Centre for Mental Health published a report outlining the difficulties in measuring outcomes and performance in liaison psychiatry.^2^ The authors suggest that the complexity and heterogeneity of service provision rule out a very simple, all-purpose approach to the measurement of outcomes and performance in liaison settings. Instead, they suggest a balanced score card approach to measuring outcome, in which account is taken of inputs (referrals to a service), activities and associated outputs (e.g. what is actually done), and outcome (benefits in health that result from the service outputs).

The aim of this study was to develop a simple, pragmatic typology to characterise the nature of liaison interventions delivered by a liaison service in a National Health Service (NHS) setting. Such a typology could then be used by other services to more fully describe the nature of their work, and enable more meaningful comparisons and benchmarking across liaison services in different settings.

## Method

The study was carried out at a large teaching hospital in Manchester. The hospital has 750 beds, with an emergency department (170 000 attendances per year) and several specialised units. An electronic record system is used to record all contacts with patients seen by the liaison psychiatry service. At the time of the study, the liaison service at the hospital had three components: a ward-based liaison service for adults of working age (16–64 years), a ward-based service for adults 65 years and older, and a nurse-led 24/7 h liaison service in the emergency department. All three components provided a seamless, flexible service. This study focuses on the adults of working age component which operates from 09:00 to 17:00, Monday to Friday, and has a standard to see patients within one working day of referral. This part of the liaison service is staffed by a consultant liaison psychiatrist (7 sessions), two senior trainees (ST4–6) and one junior trainee (ST3).

### Developing the typology for liaison interventions

All the electronic records for consecutive referrals to the liaison service for a 2-month period between 1 April 2013 and 31 May 2013 were examined. We considered the reason for referral to the service, the free text in the electronic record describing the clinical assessment, and additional entries (if present) describing subsequent contacts and interventions, plus our own clinical knowledge of the patient in question. We used a modified open-card sorting method to group referrals according to different kinds of service interventions.^3^ We developed seven distinct, definable categories, which captured the main clinical intervention: (1) diagnosis/formulation; (2) assessment of risk; (3) medication management; (4) management of disturbed behaviour; (5) assessment of mental capacity; (6) treatment of non-psychosis; and (7) treatment of psychosis.

These preliminary categories were then further refined after discussion with senior consultant colleagues and presentation at a Psychiatric Liaison Accreditation Network (PLAN) 1-day meeting, a Liaison Psychiatry Young Consultants' and Trainees' meeting and the preliminary findings from a working group set up by the Liaison Faculty of the Royal College of Psychiatrists to develop an outcome framework for liaison psychiatry. The two treatment categories (treatment of non-psychosis and treatment of psychosis) were collapsed into one category of ‘treatment’ and four other categories were added: assessment and treatment under the Mental Health Act 1983;^4^ brief psychological interventions; providing guidance/advice; and signposting/referring on. Thus, the final ten categories are:

1. assessment and diagnosis/formulation: joint working with medical or surgical teams to establish a diagnosis following uncertainty regarding symptom presentation
2. providing guidance/advice: verbal or written guidance or advice provided by the liaison team about common psychological problems, lifestyle changes or alcohol consumption
3. signposting/referring on: the individual is given information about other relevant statutory or non-statutory agencies, or referred to another service by a liaison team member
4. assessment and management of risk: the assessment and judgement of an individual's immediate risk of self-harm or harm to others; the assessment is usually followed by a management plan tailored to the severity and nature of current risk – risk changes with time, so it is not uncommon for multiple assessments of risk to be carried out
5. assessment of mental capacity: the assessment of capacity to consent to a therapeutic procedure or post-discharge placement, or ability to self-discharge
6. assessment and treatment under the Mental Health Act: the use of the Mental Health Act^4^ in the general hospital setting to detain an individual for assessment or treatment of their mental health problems
7. medication management: a consultation about psychotropic medication which requires stopping, restarting, switching or adjusting because of physical health problems; such decisions usually entail expert knowledge regarding the use of psychotropic drugs in physically unwell patients
8. management of disturbed behaviour: the active management of a patient with disturbed behaviour as a consequence of some form of mental health problem while in the general hospital setting
9. brief psychological interventions: the treatment of an individual's psychological problems using a brief psychological intervention
10. treatment: the starting or continuation of treatment for a diagnosed mental health problem; the treatment is most often some form of psychotropic medication coupled with advice about management.

### Case-note review

We carried out a retrospective case review of the electronic records of consecutive referrals to the ward-based service for 12 months from 18 June 2013 to 17 June 2014. We collected the following details: age; gender; health district of origin; the number of face-to-face contacts carried out by the team; the duration of time the team had ongoing contact with the patient (days); the reason for referral; psychiatric diagnosis using clinical judgement according to ICD-10 criteria;^5^ whether the Mental Health Act^4^ was employed; and disposal.

We assigned each patient seen by the service to one of the ten intervention categories. The ten criteria are not mutually exclusive, but for our main analysis we focused on the principal intervention by the team for each individual referral (i.e. the type of intervention which had taken up the greatest proportion of liaison input). Assignment was carried out on a consensus basis within the team.

We used face-to-face contacts to estimate the workload of the service. The minimum work involved in each face-to-face assessment includes the following: going to the hospital ward; reviewing the patient's notes; speaking to a member of nursing staff to obtain an update regarding the patient's progress; finding a private room to interview the patient; interviewing and assessing the patient; feeding back to nursing staff; writing a summary of the contact with specific advice in the medical notes; and entering a more detailed assessment on the mental health trust's electronic record system. Most face-to-face contacts usually, but not always, involve discussion with the medical or surgical team involved in the patient's care.

There are many other aspects of liaison work that are not captured by ‘face-to-face’ contact activity, including attendance at multidisciplinary meetings, telephone calls, liaising with other health professionals, relatives, carers, pharmacy, writing letters, writing reports, providing education and training, service evaluation, development and management of services, etc. However, it is very difficult to reliably record all this other activity, so we used ‘face-to-face’ contacts as a proxy measure for overall workload.

Where the data are normally distributed, summary scores are presented as means and standard deviations. The service activity data were not normally distributed, so summary scores are presented in the form of medians and interquartile ranges (IQRs). Kruskal–Wallis tests were used to compare the continuous activity data.

We determined that as the study was concerned with service evaluation, it was not necessary to seek approval from a research ethics committee. Only clinicians working for the liaison service, who had been involved in direct clinical patient care, had access to patients' electronic records (in other words, the entries the clinicians had written themselves). All clinical material included in this report has been anonymised and all identifying features removed.

## Results

The service received 344 referrals from 18 June 2013 to 17 June 2014. The average age of people seen was 47.7 years (s.d. = 15.1), and 184 (53.5%) were female. At any one time, between 8 and 15 patients were under review by the team.

Two hundred and twenty-seven (66.0%) referrals were from medical wards, 59 (17.2%) from surgical wards, 31 (9.0%) from the women's hospital and 23 (6.7%) from the critical care unit. One referral came from the eye hospital and a further three were from the liaison for older adults service of patients who were over 65 years old but who were best managed by the adults of working-age team.

Approximately half of the referrals (*n* = 168, 48.8%) involved people who lived in the locality of the hospital. The other half involved people from 18 different health districts across the north of England, and 17 patients were homeless.

Just under half of the people referred received a single assessment (*n* = 157, 45.6%). A quarter of people were seen between two and four times (*n* = 96, 27.9%), 20.1% (*n* = 69) were seen between five and ten times and a small number of people required more than ten contacts (*n* = 22, 6.4%). The total number of face-to-face contacts was 1259 for the 12-month period, with the average number per patient at 3.7 (s.d. = 4.6). The small number of patients who required more than ten contacts accounted for nearly a third of the total workload of the service (*n* = 373 face-to-face contacts, 29.6%). The mean length of duration under the care of the liaison team was 10.8 days (s.d. = 20.5), with a range of 1–180 days.

One hundred and eighty-seven patients (54.4%) were seen on at least two occasions by the team and of these, 167 (48.5%) were seen with at least 7 days between each assessment.

Table 1 shows the main mental health diagnoses of people referred to the service according to the number of referrals and the number of face-to-face contacts carried out by the team.

**Table 1:** The activity of the liaison team according to psychiatric diagnosis

	Referrals	Face-to-face contacts	Face-to-face contacts	Time in contact with service, days
		
Type of clinical problem	*n* (%)		Median (IQR)	
Depression	135 (39.2)	506 (40.2)	2 (1–5)	7 (1–14)

Schizophrenia	51 (14.8)	288 (22.9)	3 (1–7)	7 (1–14)

Bipolar affective disorder	25 (7.3)	98 (7.8)	3 (1–4.5)	4 (1–10)

Delirium	23 (6.7)	84 (6.7)	2 (1–4)	5 (1–14)

Substance misuse	16 (4.7)	58 (4.6)	1.5 (1–2)	2 (1–7)

Medically unexplained symptoms	9 (2.6)	47 (3.7)	3 (1–7.5)	3 (1–34)

No current mental health problem	25 (7.3)	40 (3.2)	1 (1–1.5)	1 (1–4)

Personality disorder	13 (3.8)	36 (2.9)	1 (1–3.5)	1 (1–9.5)

Korsakoff syndrome	12 (3.5)	27 (2.1)	1.5 (1–2)	4 (1–17)

Adjustment disorder	10 (2.9)	21 (1.7)	1 (1–2)	1 (1–4.75)

Anxiety disorder	12 (3.5)	19 (1.5)	1 (1–1.75)	1 (1–1.75)

Eating disorder	5 (1.5)	22 (1.7)	3 (1.5–8)	7 (2–14.5)

Intellectual disability	1 (0.3)	1 (0.1)	1	1

Asperger syndrome	1 (0.3)	1 (0.1)	1	1

Dementia	6 (1.7)	11 (0.9)	1 (1–2.75)	1 (1–6.25)

Total	344 (100)	1259 (100)	2 (1–5)	4 (1–13)

IQR, interquartile range.

Depression accounted for approximately 40% of referrals and 40% of the face-to-face workload. Schizophrenia and bipolar affective disorder accounted for 22% of referrals but 30% of the workload. There were significant differences between diagnoses for both number of contacts (*P* = 0.003) and duration of contact (*P* = 0.009) using the Kruskal–Wallis test. The groups with the largest median number of contacts were individuals with schizophrenia, bipolar affective disorder and somatoform disorders, with a median of 3 contacts.

Table 2 shows the service activity for each principal intervention category. Of the 344 patients referred to the service over 12 months, 4 people declined any help from the service, so were not allocated to an intervention. Of the remainder, the most frequently employed intervention was diagnosis (*n* = 112 patients, 32.6%), followed by medication management (*n* = 57 patients, 16.6%), then risk assessment and treatment (both 56 patients 16.3% each), followed by management of behavioural disturbance (*n* = 28 patients, 8.2%). Although assessment or treatment using the Mental Health Act^4^ only accounted for 23 patients (6.7% of referrals), this work involved the highest median number of contacts (7, IQR 3–12), and a total number of 230 (18.3%) face-to-face contacts. There were no patients allocated to ‘brief psychological interventions’, ‘signposting/referring on’ or ‘providing advice/guidance’.

**Table 2:** Referrals and service workload according to the principal intervention categories

	Referrals *n* (%)	Number of contacts Median (IQR)	Total number of contacts *n* (%)	Time in contact with the service, days Median (IQR)
Assessment and diagnosis/formulation	112 (32.6)	1.5 (1–3)	316 (25.1)	3 (1.0–8.0)

Assessment and management of risk	56 (16.3)	1.0 (1–2)	139 (11)	1 (1–6.5)

Assessment of mental capacity	8 (2.3)	1.0 (1–1.8)	10 (0.8)	1 (1–6.3)

Mental Health Act 1983 assessment	23 (6.7)	7.0 (3–12)	230 (18.3)	14 (6–30.0)

Medication management	57 (16.6)	3.0 (1–5)	208 (16.5)	6 (1–14.0)

Management of disturbed behaviour	28 (8.1)	2.0 (1–5)	105 (8.3)	5 (1–8.8)

Treatment	56 (16.3)	2.5 (1–6)	245 (19.5)	7 (1–21.0)

No engagement	4 (1.2)	1.0 (1–2.5)	6 (0.5)	1 (1–10.8)

Total	344	2.0 (1–5)	1259	4 (1–13)

IQR, interquartile range.

There were significant differences between principal intervention groups for both number of contacts (*P*<0.001) and duration of contact (*P*<0.001) using the Kruskal–Wallis tests (Table 2). The Mental Health Act group had the greatest number of contacts (median 7), followed by medication management (median 3), treatment (median 2.5) and management of disturbed behaviour (median 2), with similar ordering of groups for workload.

Approximately half the patients seen by the service (*n* = 164, 47.7%) received a therapeutic intervention as the principal intervention of the team, which included treatment of a mental health problem, medication management, management of disturbed behaviour, or detention to hospital under the Mental Health Act^4^ for either assessment or treatment.

### Interventions

Table 3 provides examples of patients assigned to each of the different liaison interventions, together with the reason for referral, their psychiatric diagnosis and the degree of contact with the service. Two examples are given for each intervention to illustrate how the liaison typology was used in clinical practice.

**Table 3:** Examples of patients allocated to the different liaison intervention categories

Liaison intervention	Age range and gender	Reason for referral	Medical condition	Psychiatric diagnosis	Outcome
Assessment and diagnosis- formulation	16–20 years, female 60–69 years, female	Developed ‘fits’ on post surgery ward round Psychogenic vomiting	Admitted for tonsillectomy	Non-epileptiform attack disorder	Explanation of nature of symptoms, engagement of patient with biopsychosocial formulation Referral for out-patient psychological treatment Request for urgent scan which showed hydrocephalus and brain metastases Patient transferred to neuroscience centre
			Vomiting query cause Known history of bipolar affective disorder	Intracranial pathology suspected as GCS low	

Assessment and management of risk	60–70 years, male	Lacerated throat with Stanley knife	Severe trauma to neck requiring tracheostomy and later PEG	Depressive disorder	Ongoing risk assessment while in-patient with treatment Principal decision involved judgement concerning discharge to home treatment rather than in-patient psychiatric bed Ongoing risk assessment while in hospital with discharge to HTT
	40–50 years, male	Stabbed self in chest	Surgery to repair multiple wounds to chest wall and explore damage to heart	Adjustment disorder	

Assessment of mental capacity	30–40 years, female	Refusing investigations and treatment Thought by staff to have paranoid ideas Refusing medical treatment Known diagnosis of schizophrenia	HIV multifocal leuco- encephalopathy	Delirium	Determine not to have capacity Best interests meeting arranged to determine further medical treatment plan
	50–59 years, female		Chest infection	Delirium, schizophrenia	Determine not to have capacity Best interests meeting arranged

Mental Health Act assessment	50–60 years, female	Paranoia	Confusion caused by chest infection which was treated Admitted for investigation of confusion No medical cause found	Schizophrenia	Detained under the Mental Health Act as refusing psychiatric treatment and considered to be at risk to self through serious neglect Transferred to in-patient psychiatric bed Detained under the Mental Health Act as trying to leave ward naked Treated on ward for 10 days while awaiting psychiatric bed Responded to treatment so Section 2 rescinded and discharged to local CRT
	40–50 years, female	Confusion Visual and tactile hallucinations		Hypomania, although very organic presentation	

Medication management	40–50 years, female 50–60 years, male	Lithium stopped Query about treatment Clozapine stopped while in hospital for 10 days	Diabetes insipidus secondary to lithium Admitted to ITU with severe chest infection Noted to have ejection fraction of 30%	Bipolar affective disorder	High risk of relapse so started on valproate Dose titrated up and discharged to CMHT
				Schizophrenia	Physical condition improved and ejection fraction improved Initial bedside measure was probably inaccurate Clozapine restarted and re-titrated after risks and benefits discussed with patient

Management of disturbed behaviour	40–49 years, male 40–49 years, female	Repeated self- harm on ward	Epilepsy	Personality disorder	Liaison team worked with staff to de-escalate behaviour and plan safe discharge with appropriate follow-up Liaison team worked with staff to establish a clear management plan and defuse negative staff attitudes
		Leaving ward to drink alcohol and also drinking from alcohol gel dis- pensers on ward	Alcoholic liver disease	Personality disorder	

Treatment	40–50 years, male	Confusion – query symptoms psychogenic, not eating or drinking Postnatal risk assessment	Confusion and weakness of unknown cause	Psychotic depression	Treatment with antidepressants and antipsychotics started immediately Patient discharged to HTT
	30–40 years, female		Normal full-term delivery History of bipolar affective disorder	Hypomania	Treatment started immediately Condition settled within 5 days and patient discharged safely with baby, with support from local CRT

CMHT, community mental health team; CRT, crisis resolution team; GCS, Glasgow Coma Scale; HTT, home treatment team; ITU, intensive therapy unit; PEG, percutaneous endoscopic gastrostomy.

## Discussion

Previous published work on the evaluation of liaison psychiatry services has predominantly focused on the following areas: activity and nature of referrals,^6,7^ the complexity of patient problems,^8^ the professional mix and staffing of services,^9–11^ training^12^ and quality outcomes.^13^ There has been relatively little work which has addressed directly either the ‘outputs’ or the outcome of liaison services. It is the outputs of liaison work that this study attempted to address, as we argue that until the outputs can be better characterised, it is difficult to determine appropriate outcome measures.

In the late 1980s, Huyse and colleagues^14^ developed a schema to operationalise the recommendations given by liaison specialists to referring teams. This was an important development, but the measure was intended for mainly one-off consultations, where advice only was provided. Our intention was to develop a typology which was practical and easy to apply in a clinical liaison setting, which could provide an indication of the type of work undertaken by a liaison team. By using the typology, we were able to determine that approximately half the workload of our service involves the treatment on medical and surgical wards of patients with mental health problems, the management of behavioural disturbance and medication management. The treatment provided includes the management and treatment of patients detained under the Mental Health Act to the acute hospital, which accounts for a fifth of the workload of the service. A third of the work involves risk assessment and diagnosis.

Approximately half of all the patients referred to our service were seen on at least two occasions, 7 days apart. This suggests that some form of paired assessment of outcome would be feasible for 50% of all referrals we receive. The different intervention categories allow us to specify in Sharpe's terms^1^ what we are seeking to achieve for each kind of intervention. For example, in the ‘treatment’ and ‘management of behavioural disturbance’ categories, we would expect to see an improvement in patients' mental health, if rated by an appropriate measure. However, if the intervention primarily involves ‘medication management’, the intention for patients assigned to this category is to prevent relapse by judicious switching or other appropriate action regarding the patient's psychotropic medication. The aim in this case for any kind of paired measurement would be to maintain stability, rather than see positive improvement.

A major problem in the clinical evaluation of liaison services has been their diversity, with different mixes of staff and different settings, including emergency department work, acute hospital work and out-patient treatment. As such, liaison services are likely to vary dramatically in their intervention profiles. However, if we delineate and describe the type of interventions which are delivered by a service, it enables us to look inside the ‘black box’ and convey to our general hospital colleagues, managers and commissioners what we actually provide, and what we are hoping to achieve.

Three of the new categories, which were suggested by liaison colleagues (brief psychological intervention, sign-posting/referring on, providing guidance and advice) were not relevant for our own service, but may be important interventions for other kinds of liaison services.

The number of referrals did not capture the workload of our service, which was better provided by the number of face-to-face contacts for each referral, as some patients required intensive input from the team, particularly those detained under the Mental Health Act.^4^ The actual number of face-to-face contacts by the adults of working-age service was very similar to the number of contacts for the team covering older adults at the same hospital. The older adult team received many more referrals during the same period, but had fewer contacts per patient than the adults of working age team.

Risk assessment and diagnosis are categories that could be assumed to involve one-off assessments by a liaison service; however, referrals involving these two kinds of intervention required a median of two to three face-to-face contacts. Many of the risk assessments involved patients who had been admitted following near-fatal self-harm, and required ongoing assessment of risk as their physical condition improved. Diagnosis also required more than one assessment in many cases, especially if there were complex issues.

Using the data from the intervention categories, together with case illustrations, helps to sketch out the work of the service. A picture emerges of a service which provides assessment, treatment and management of patients with complex and high-risk mental health problems in the setting of an acute hospital. For at least half the patients, the liaison service operates almost like a cross between a crisis resolution service and an in-patient psychiatric unit (but in the setting of the acute hospital). The anonymised case descriptions illustrate the bi-directional interplay between psychological and physical problems, which can be hard to convey using numerical or quantitative data.

The intervention categories have recently been incorporated into the Framework for Routine Outcome Measurement in Liaison Psychiatry (FROM-LP),^15^ and have been termed the IRAC scales (Identify and Rate the Aim of the Contact). It is suggested they are used in conjunction with a variety of recommended outcome measures, depending on whether the patient is seen on one, or more than one, occasion.

There are inherent limitations in any single-site observational study, and our results are not generalisable to other liaison services. The study was conducted in a large inner-city teaching hospital, and the patients referred to the service may not be typical of those seen by other liaison services. Nevertheless, the methods we employed can be reproduced by any liaison service and will provide a way of comparing and benchmarking services. We believe this study provides preliminary support for the new guidance from the Faculty of Liaison Psychiatry of the Royal College of Psychiatrists regarding outcome measurement.
